# An Overview of Epigenetic Methylation in Pancreatic Cancer Progression

**DOI:** 10.3389/fonc.2022.854773

**Published:** 2022-02-28

**Authors:** Yuhao Zhao, Mao Yang, Shijia Wang, Sk Jahir Abbas, Junzhe Zhang, Yongsheng Li, Rong Shao, Yingbin Liu

**Affiliations:** ^1^ Department of Biliary-Pancreatic Surgery, Renji Hospital Affiliated to Shanghai Jiao Tong University School of Medicine, Shanghai, China; ^2^ State Key Laboratory of Oncogenes and Related Genes, Shanghai Cancer Institute, Shanghai, China; ^3^ Shanghai Key Laboratory of Biliary Tract Disease Research, Shanghai, China; ^4^ Department of Pharmacology, Shanghai Jiao Tong University School of Medicine, Shanghai, China

**Keywords:** Epigenetics, Pancreatic cancer, DNA methylation, Histone methylation, RNA methylation

## Abstract

Over the past decades, the aberrant epigenetic modification, apart from genetic alteration, has emerged as dispensable events mediating the transformation of pancreatic cancer (PC). However, the understanding of molecular mechanisms of methylation modifications, the most abundant epigenetic modifications, remains superficial. In this review, we focused on the mechanistic insights of DNA, histone, and RNA methylation that regulate the progression of PC. The methylation regulators including writer, eraser and reader participate in the modification of gene expression associated with cell proliferation, invasion and apoptosis. Some of recent clinical trials on methylation drug targeting were also discussed. Understanding the novel regulatory mechanisms in the methylation modification may offer alternative opportunities to improve therapeutic efficacy to fight against this dismal disease.

## Introduction

Pancreatic cancer (PC) is a type of tumor with high malignancy and aggressiveness. It is ranked the 8th leading causes of cancer death in the world in 2020 and its 5-year survival rate is less than 7% (1, 2). Although surgical radical resection remains the mainstay of PC treatment (3, 4), most PC patients are diagnosed at an advanced stage and miss the opportunity for surgery as they frequently appear to be atypical symptoms (5, 6). Even for the patients that undergo surgical resection, the rate of recurrence and death after surgery is particularly high. In the early diagnosis, the widely applied screening methods such as the detection of tumor marker CA19-9 and imaging yield minimal benefit as the measurement sensitivity does not give rise to the levels different from normal (7, 8). As a result, we need to further investigate the mechanisms of PC development to identify more molecules that can be detected at early stages to improve early diagnosis.

**Figure 1:**
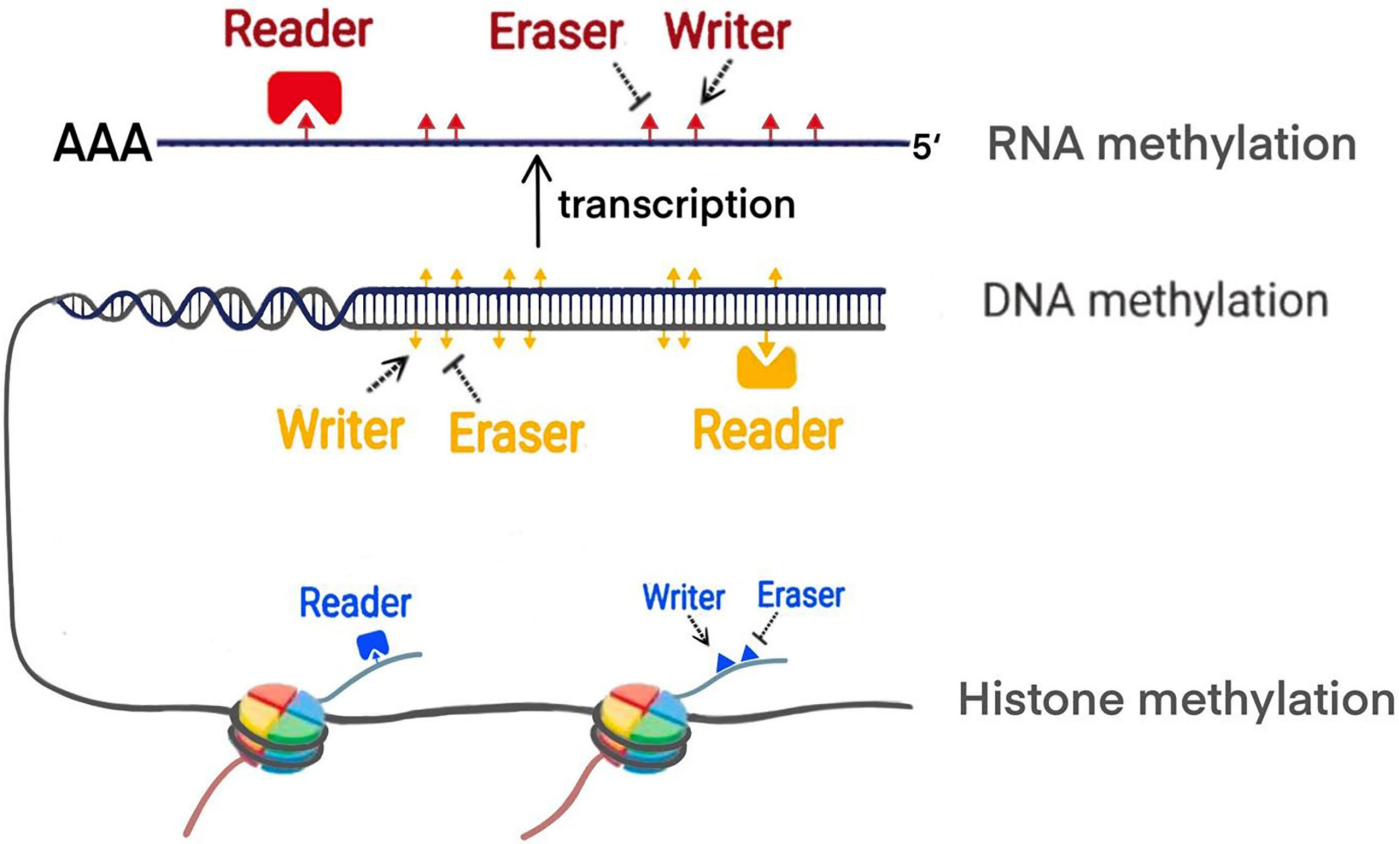
The modification of methylation by writer, eraser and reader.

Increasing evidence shows that PC is associated with polygenic lesions, which include gene mutations and epigenetic modifications. Epigenetics proposed by Waddington, refers to reversible and heritable changes in gene function instead of the sequence alternations (9). Methylation modification are one of the main manifestations of epigenetics. The previous studies demonstrated that the methylation process is mainly regulated by writer, eraser and reader (10) (**Figure 1**). Writer represents methyltransferase which can transfer the methyl group to specific site of DNA, RNA and histone. For example, DNA methylation mostly occurs in cytosine-phosphate-guanine (CpG) islands. In histone methylation, both lysine and arginine residues can be catalyzed. Various modifications of RNA methylation have been found, including N6-methyladenosine (m6A), 5-methylcytosine (m5C), andN1-methyladenosine (m1A). Eraser refers to demethylases which remove the methyl group. Reader is a class of proteins that are able to recognize methylation mark by their distinct domains and induce different biological functions. Up to date, the widespread application of methylation biomarkers detection and the emergence of epigenetic drug targets has brought new possibilities for the diagnosis and treatment of PC. Future therapy of PC will expectedly focus on some new targets revealed including epigenetic regulatory molecules. In this review, we particularly focus on the discussing the mechanism of the methylation modifications in PC from DNA methylation, histone methylation and RNA methylation modifications. Furthermore, we update recent clinical trials that target epigenetic methylation molecules.

## DNA Methylation

DNA methylation, the most well-studied epigenetic modifications, often precedes before somatic cell mutation and occurs in early tumorigenesis. In the process of DNA methylation, S-adenosyl-L-methionine (SAM) provides the methyl group, which is transferred to specific site of DNA, including 5-methylcytosine, N6-methyladenine and 7-methylguanine. Methylation occurs mostly at the cytosine-C5 of the CpG dinucleotides which exists in two forms: CpG islands and CpG island shores. CpG islands, a region of the CpG dinucleotides cluster, are approximately located in 60% of gene promoters. CpG island shores, close to CpG islands, refer to a region that CpG dinucleotides disperse. In normal cells, only 5% of the CpG islands are methylated in the promoters; in contrast, all CpG island shores are usually methylated (11). DNA methylation modification does not alter the sequence or the composition of nucleotides; instead, it participates in the regulation of gene expression. For example, aberrant methylation of CpG islands in the promoter leads to downregulation of a variety of gene expression through the interaction with methylation-binding proteins. These proteins may act as transcriptional repressors to block the binding of the transcription factors, resulting in gene silencing.

Abnormal DNA methylation (hypermethylation and hypomethylation), mainly in CpG islands, is closely related to tumor development including PC. Overexpression of DNA methyltransferase (DNMT) leads to hypermethylation of gene promoters. The experimental evidence has shown that the genes with hypermethylation are tumor suppressors and are diminished or silenced due to hypermethylation in PC. DNA hypomethylation can be found not only in CpG islands, but also in CpG island shores when DNMT is inactivated, or demethylases are overexpressed. Currently, the most common genes with hypomethylation are oncogenes whose expression or activity is upregulated in tumor progression (12). Therefore, growing evidence has revealed on the specific mechanisms of methyltransferase (writer), demethylase (eraser) and DNA binding protein (reader) in the regulation of abnormal methylation during the development of PC (**Table 1**).

**Table 1:** Major groups of DNA methylation regulators in PC.

Methylation enzymes	Family	Alias	Function in PC
Writer	DNMTs	DNMT1	pro-PC
		DNMT2	unclear
		DNMT3a	pro-PC
		DNMT3b	pro-PC
		DNMT3l	unclear
Eraser	TETs	TET1	anti-PC
		TET2	unclear
		TET3	unclear
Reader	MBDs	MeCP2	pro-PC
		MBD3	anti-PC
	SRA domain-containing proteins	UHRF1	pro-PC
		UHRF2	unclear
	Methyl-CpG binding zinc fingers	Kaiso	pro-PC
		KLF4	anti-PC

### DNA Methyltransferase (Writer)

The degree of methylation, which is catalyzed by DNMT, is related to the activity and expression of DNMT. DNMT expression is closely associated with the prognosis of PC. DNMTs include DNMT1, DNMT2, DNMT3a, DNMT3b, DNMT3l and their isoforms.

DNMT1 acts as the most important DNMT for maintaining the methylation of genes (13). It has three domains, the catalytic domain at the C-terminus, the target region recognized by certain proteins at the N-terminal part and the unknown region. DNMT1 is overexpressed in PC and its expression gradually increases with the transformation process from normal tissue, precancerous lesions to PC, indicating that the high expression of DNMT1 is associated with poor prognosis in patients (14). High DNMT1 expression is closely related to neural infiltration, tumor differentiation and TNM staging in PC, suggestive of a potential target for treatment of PC (15). Also, several studies showed that DNMT1 may regulate a variety of downstream genes to promote PC cell proliferation, migration and invasion as well as self-renewal of PC stem cells, such as suppressing the expression of Cyclin-dependent kinase inhibitors (CKIs) (16–18). Transfecting PC cells with siRNA DNMT1 reveals a significant decrease in cell proliferation and migration (19). In addition, n-butylidenephthalide (n-BP), a novel DNMT1 inhibitor, suppresses PC cell proliferation and blocks PC cells in G0/G1 phase (20). At present, phase I/II clinical trials of other DNMT1 inhibitors (Azacitidine, Decitabine) are ongoing (NCT01845805, NCT02959164) with the expectation of curing PC. DNMT3a and DNMT3b are the major *de novo* methylation enzymes, that affect the expression of target genes by regulating the level of DNA methylation (21). DNMT3a is highly expressed in PC and is closely associated with poor prognosis (22). Downregulation of DNMT3a in PC cell lines enhances their chemosensitivity to gemcitabine and oxaliplatin. Knocking out of DNMT3a inhibits cell proliferation, induces cell cycle arrest, and promotes apoptosis by decreasing cyclin D1 expression (22, 23). Studies about DNMT3b on PC are less reported compared to DNMT3a. For example, siRNA DNMT3b treatment of PC cells inhibit cell proliferation, while overexpression of miR-29b which targets DNMT3b promotes cell apoptosis (24). As a result, both DNMT3a and DNMT3b may become new targets for PC therapy. Neither DNMT2 nor DNMT3l possesses methyltransferase activity. However, DNMT3l is essential for *de novo* methylation, which interacts with DNMT3a and DNMT3b, stimulating their enzymatic activities (25, 26).

### DNA Demethylase (Eraser)

DNA demethylation processes are divided into active demethylation and passive demethylation. Active demethylation is performed by demethylation enzymes which remove methylation marks, regulate gene expression and express different biological functions (27). Currently, the demethylases identified include the ten-eleven translocation family (TETs, TET1, TET2 and TET3) and ALKBH1 (27). Passive demethylation is a process which terminates due to the lack of DNMT1. In general, compared to DNMT, demethylase is less reported in PC. Several other demethylases need to be investigated apart from the TET1.

### TETs

TETs were not recognized as demethylases until 2009. They convert 5-methylcytosine(5mC) to 5-hydroxymethylcytosine (5hmC), further generating 5-formylcytosine (5fC) and 5-carboxycytosine (5caC) to complete the demethylation process (28). TET1 is a 5mC hydroxylase that has been defined as a tumor suppressor in PC due to its low expression (29, 30). The overall survival of PC patients with low TET1 levels is shorter than those with high TET1 levels. TET1 is proved to suppress epithelial-mesenchymal transitions (EMT) in PC by inhibiting the Wnt signaling pathway (29). Other members of this family have similar function as TET1, but their mechanisms remain to be clarified in PC.

### DNA Binding Protein (Reader)

Reader is a class of proteins or domains in the DNA methylation process, which can combine with different types of methylation modifications and interpret different biological functions. There is a mutual regulation between ‘writer’ and ‘reader’ (31). Familiar readers are divided into three categories, including the methyl-CpG-binding domain family (MBDs), SRA domain-containing proteins and Methyl-CpG binding zinc fingers (32–34).

### MBDs

MBDs are key members in determining the transcriptional status of the epigenome, which bind to methylated CpG dinucleotides and exhibit various transcriptional regulatory effects (32). Up to date, there are eleven known members of the MBDs consisting of MeCP2, MBD1-6, SETDB1/2 and BAZ2A/B. But their roles in in PC are not well understood except a few members (35). MeCP is the first identified MBD domain-containing protein and considered to be an oncogene in PC, promoting EMT in PC cells (36). However, MBD3 plays a suppressive role in PC. Downregulation of MBD3 promotes the proliferation, migration and invasion of PC cells (37). Whether the opposite activities of MeCP2 and MBD3 present in PC are required to be further clarified. Nevertheless, both of them may become new targets for future treatment of PC.

### SRA Domain-Containing Proteins

The SRA domain-containing proteins are another class of readers that contain SRA domain and are bound to the DNA hemi methylated regions (38). It consists of two main members: Ubiquitin-like with PHD and RING finger domain 1 (UHRF1) and Ubiquitin-like with PHD and RING finger domain 2 (UHRF2) (39). UHRF1 plays multiple roles in DNA methylation, which maintains DNA methylation during replication and is considered as a pivotal protein for integrating epigenetic information (40). UHRF1 is highly expressed in variety of cancers and is associated with tumorigenesis, progression and invasion (41). UHRF1 mediates the silencing of PC suppressor genes and regulates the proliferation, metabolism and metastasis of PC cells through the UHRF1/SIRT4 axis (42). In addition, it can also regulate PC cell by other pathways (43).

### Methyl-CpG Binding Zinc Fingers

Methyl-CpG binding zinc fingers are the third class of readers, which mainly binds to DNA methylation regions through c-terminal zinc finger motifs. This family has developed rapidly over the past few years, and there are eight members, including Kaiso, ZBTB4, ZBTB38, ZFP57, KLF4, EGR1, WT1, CTCF (44). However, studies of this family on PC are comparatively less and more worthy of exploration. Kaiso is the first member of the family that binds to both methylated and non-methylated regions of DNA, and its role in tumors may vary. Kaiso is overexpressed in aggressive and metastatic PC tissues, and its nuclear expression increases with aggressiveness and lymph node positivity (45). The underlying mechanisms involved in PC remain unclear. Only KLF4 is relatively well studied in PC and is similar to Kaiso in that it binds to both DNA methylated and DNA non-methylated regions (46). Nowadays, KLF4 has been reported to play either a promotive or inhibitory role in tumors, in which it is mostly considered as a tumor suppressor in PC. KLF4 limits PC metastasis by negatively regulating CD44, which provides theoretical evidence for KLF4-regulated therapy in advanced PC patients (47).

## Histone Methylation

Histone modification is one of the most important post-translational epigenetic modifications, that can regulate multiple genetic events including transcription, DNA repair by influencing chromatin structure, recruiting remodeling enzymes or transcription complexes. Abnormal changes in a variety of histone modifications may promote the progression of PC. Histone modifications includes acetylation, methylation, phosphorylation, ubiquitination, and small ubiquitin-like modifier (SUMO). Histone methylation plays a primary role in regulating gene transcription.

Post-translational methylation in histone tails usually occurs in the lysine (K), and arginine(R) residues of histone H3 and H4. Residues of these amino acid can be mono-, di-, and trimethylated (only in lysine residues) to activate or inhibit gene transcription, depending on the specific situation (such as methylation site, state and number). For example, H3K4, H3K27, H3K36, H3K79and H4K12 in lysine residues largely promote transcriptional activation, while H3K7, H3K9, H3K56, H4K5 and H4K20 inhibit gene transcription (48). In arginine residues, H3R8, H3R17 and H4R3 activate transcription of downstream genes (48). Like DNA methylation, methyl group is dynamically added by methyltransferases-writers, removed by demethylase-erasers, and interpreted by proteins with methyl binding motifs-readers. These readers recognize histone methylation and help histone writers and erasers to locate appropriately. Different histone methylation sites are catalyzed by specific enzymes (**Table 2**). The balance between histone methylation and demethylation regulated by these enzymes has been shown to affect embryonic development and various physiological functions. Structural abnormalities or functional defects of these enzymes lead to a series of serious diseases (49). Numerous studies have confirmed that histone methylation has an impact on the progression of PC and related methyltransferases and demethylases inhibitors may be used as potential means to treat PC.

**Table 2:** Major groups of histone methylation regulators in PC.

Methylation enzymes	Family		Alias	Function in PC
Writer	KMTs		SMYD3	pro-PC
			EZH2	pro-PC
	PRMTs		PRMT1	pro- PC
			PRMT5	pro- PC
Eraser	KDMs	LSDs	KDM1	pro- PC
		JMJDs	KDM2B	pro- PC
			KDM3A	pro- PC
			KDM4A	anti- PC
			KDM4B	pro- PC
			KDM4D	unclear
			KDM5A	unclear
			KDM6B	anti- PC

### Lysine Methyltransferase (Writer)

Histone lysine methylation is catalyzed by lysine methyltransferases (KMTs) in the presence of SAM as the methyl donor. The two major writers, KMT3E (SET and MYND domain-containing protein 3, SMYD3) and KMT6 (enhancer of zeste homolog 2, EZH2) are known to function in PC.

### SMYD3

SMYD3 belongs to the SET and myeloid-Nervy-DEAF-1 (MYND)-domain family that catalyzes lysine 4 of histone H3(H4K5). SMYD3 has been widely explored because of its increased expression in many types of cancer, particularly those driven by the Ras signaling activation (50). SMYD3 is upregulated in PC and indicates a poor prognosis. Moreover, PC with a high expression of SMYD3 also has high caspase-3 and MMP-2 expressions (51). Decreased SMYD3 expression impairs cell growth and metastasis of PC *in vitro*. The MMP-2 mRNA and protein expressions are also downregulated in SMYD3 knock-down cell lines, but the expression of caspase-3 has not significantly changed. SMYD3 could be a candidate therapeutic target against tumorigenesis because of SMYD3 inhibitors discovered. There is a reported small molecule inhibitor targeting SMYD3 called piperidine-4-formamide-acetanilide compound, BCI-121 (52). It is a small molecule inhibitor that significantly reduces SMYD3 activity and inhibits proliferation in PC cell lines with SMYD3 overexpressed. However, this inhibitor has not been approved for clinical trials yet.

### EZH2

EZH2 is the functional subunit of polycomb repressive complex 2 (PRC2), that epigenetically represses the expression of tumor suppressor gene through trimethylating lysine 27 of histone H3(H3K27) in various cancer types. High expression of EZH2 is associated with PC (53). FBW7, an E3 ubiquitin ligase of EZH2, downregulates EZH2 through ubiquitination and degradation in PC cells. Activated CDK5 kinase can catalyze the EZH2 phosphorylation that is required for FBW7-mediated EZH2 degradation. Low expression of FBW7 causes an aberrant accumulation of EZH2 and induces tumorigenesis in PC (53). Non-coding RNAs can recruit EZH2 to modify histone H3 lysine 27 trimethylation (H3K27me3) of downstream target genes. For example, long non-coding RNA (lncRNA) BLACAT1 inhibits CDKN1C expression *via* EZH2-induced H3K27me3 and promotes proliferation, migration, and aerobic glycolysis of PC cells (54). Highly-expressed EZH2 and low expressed miR-139-5p are detected in PC tissues and their expressions are associated with poor prognosis. Downregulation of EZH2 and upregulation of miR-139-5p impede EMT and lymph node metastasis (LNM) in PC cells. Mechanistically, EZH2 suppresses the expression of miR-139-5p through upregulating H3K27me3 (55). 3-Deazaneplanocin A (DZNeP) can reduce EZH2 and H3K27me3 expression. It shows that DZNeP/gemcitabine combination can significantly increase the apoptosis rate of PC cells, which seems to be promising anticancer reagents (56).

### Lysine Demethylase (Eraser)

Histone lysine demethylases (KDMs) catalyze the removal of methyl groups on histone lysine residues, which is a reversible process. Based on the mechanism of action, KDMs are classified into two families: Flavin adenine dinucleotide (FAD)-dependent and Fe (II) and 2-oxoglutarate (2OG)-dependent.

### KDM1

KDM1 is the only FAD-dependent KDM that is related to PC. The expression of two subtypes of KDM1, KDM1A (lysine-specific demethylase1, LSD1) and KDM1B (lysine-specific demethylase2, LSD2) are both elevated in PC tissues. The role of KDM1A in regulating PC progression is poorly understood. As for its homolog KDM1B, interfered KDM1B expression in PC cell lines reduces the cell proliferation and significantly increases the cell apoptosis (57).

### JMJD Domain-Containing Protein Family

Another type of KDMs is from Jumonji C domain-containing (JMJD) protein family which is Fe (II) and α-ketoglutarate-dependent dioxygenases. Altered activity of JMJD protein family members is emerging as a common cause of tumor progression. In the study of PC, amplification or overexpression of the H3K9/H3K36 demethylases such as KDM2B、KDM3A and KDM4 exert positive roles in PC progression. KDM6B, an H3K27 demethylase, plays as a tumor suppressor.

KDM2B acts an active factor to drive the tumorigenicity. It mediates poorly differentiated PC through two different mechanisms. Occupancy of transcriptional start sites together with polycomb group (PcG) proteins represses developmental genes which function in cell cycle progression and senescence. In co-binding with the MYC oncogene and/or the histone demethylase KDM5A, KDM2B can activate the transcription of a module of genes involved broadly in metabolic homeostasis (58).

KDM3A participates in the epigenetic upregulation of DCLK1 expression which is correlated with PC morphology (59). DCLK1 is characteristic of a morphologically distinct subpopulation of stem-like cells in PC and its expression reveals the cellular and functional heterogeneity in PC (60).

The KDM4 subfamily mainly include 4 demethylases, including KDM4A, B, C and D. They are all studied and reported to play a role in PC, except KDM4C. Regulatory factor X-associated protein (RFXAP), a key transcription factor for MHC II molecules, binds to the promoter of KDM4A and promotes its transcription, thereby demethylating histone H3K36 (61). In PC, Fisetin induces DNA damage through RFXAP/KDM4A-dependent demethylation to inhibit proliferation *in vivo* and *in vitro (* 62). KDM4B plays a crucial role in EMT process (63). It demethylates histone H3K9 to activate ZEB1 transcription (63). ZEB1 acts as an E-box binding transcription factor which is reported to epigenetically downregulate E-cadherin expression (64). High nuclear KDM4D expression in the specimens of pancreatic resection margins are significantly associated with dismal disease-free survival and can be an independent predictor of recurrence risk in PC patients (65). However, its physiological role in PC remains unknown.

It is known that oncogenic KRAS mutations can be detected in nearly all pancreatic lesions. KDM6B, the downstream of KRAS, is downregulated in PC cells with the lowest expression level in poorly differentiated PC (66). KDM6B knockdown can inhibit the expression of the CCAAT-enhancer binding protein alpha (CEBPA) gene and enhance tumor progression of PC cells both *in vitro* and *in vivo (* 66).

The study of KDM5 family needs to be deepened in PC. KDM5A is a demethylase for histoneH3K4. KDM5A epigenetically suppresses the expression of mitochondrial pyruvate carrier-1 (MPC-1) and promotes the cell proliferation through mitochondria pyruvate metabolism in PC (67).

### Arginine Methyltransferase (Writer)

Arginine residues can be methylated by protein arginine N-methyltransferases (PRMTs), which are classified as type I, II, or III enzymes according to their catalytic activity. PRMT1 from type I and PRMT5 from type II are related to PC.

### PRMT1

Approximately 90% of total arginine methylation is catalyzed by PRMT1. As for histones, PRMT1 can catalyze the methylation of arginine 3 on histone H4(H4R3), which activates gene transcription. PRMT1 reported to be highly expressed in various cancer types, as well as in PC. Elevated expression level of PRMT1 is significantly associated with poor prognosis in PC patients. Functional experiments show that PRMT1 promotes PC cell proliferation *in vitro* and *in vivo*, and induces the upregulation of the β-catenin (68). The Wnt-β-catenin signaling pathway has already been highly implicated in pancreatic carcinogenesis and progression (69).

### PRMT5

PRMT5 catalyzes the symmetrical dimethylation of arginine 8 on histone H3(H3R8) and arginine 3 on histone H4 (H4R3). Several studies show that PRMT5 plays a critical role in tumorigenesis and metastasis (70). As for PC, PRMT5 expression is highly expressed in tumor tissues. It promotes cell proliferation, migration, invasion, and EMT *via* activating EGFR/AKT/β-catenin signaling in PC cells (71). In addition, PRMT5 is proved to epigenetically suppress the promoter activity of FBW7 which controls the level of cMyc *via* ubiquitination and degradation (72). FBW7 is an E3 ubiquitin ligase that controls cMyc degradation. Mechanistically, PRMT5 post-translationally regulates c-Myc stability. Elevated c-Myc levels promote the proliferation of and aerobic glycolysis in PC cells (72). EZP015556, an inhibitor of PRMT5, is found to be effective in MTAP (a gene commonly lost in PC) negative tumors in preclinical experiments, and now there are a few clinical trials on this inhibitor ongoing (NCT03573310, NCT02783300, and NCT03614728) (73).

### Arginine Demethylase (Eraser)

Corresponding to methylation, histone demethylation can occur in arginine residues and lysine residues. However, there is a large gap in research on arginine demethylases. To date, there have been no definite reports of specific arginine demethylases (74). In general, well-balanced arginine methylation is important for cellular proliferation and differentiation. Consequently, certain enzymes such as PRTMs, catalyze arginine methylation modifications and other enzymes acting as eraser of arginine methylation may participate in the demethylation, but remain to be established.

## RNA Methylation

RNA methylation is a process that mediates RNA metabolism and gene expression. Over 150 modifications are identified in all types of RNA, in which RNA methylation is one of the most important forms of RNA modifications. These post-transcriptional RNA methylations include N6-methyladenosine (m6A), 5-methylcytosine (m5C) and N1-methyladenosine (m1A). RNA methylation can be dynamically and reversibly regulated by methyltransferase (writer), demethylase (eraser) and RNA binding protein (reader) (**Table 3**).

**Table 3:** Major groups of RNA methylation regulators in PC.

Sites	Methylation regulators	Family	Alias	Function in PC
m6A	Writer	methyltransferase complex (MTC)	METTL3	pro-PC
			METTL14	pro-PC
			WTAP	pro-PC
	Eraser	AlkB homolog proteins	ALKBH5	anti-PC
		FTO		pro-PC
	Reader	YTH structural domain proteins	YTHDF2	anti-PC/pro-PC
		IGF2BPs	IGF2BP2	pro-PC
		hnRNP family		unclear
m5C	Writer	NOL1/NOP2/Sun domain family	NSUN6	anti-PC
	Eraser	TETs		unclear
	Reader	ALYREF		unclear
		YBX1		unclear
m1A	Writer	TRMTs		unclear
		NML		unclear
	Eraser	AlkB homolog proteins	ALKBH1	pro-PC

### N6-Methyladenosine (m6A)

N6-methyladenosine (m6A) is the most abundant methylation modification of eukaryotic messenger RNA (mRNA) (75). The m6A site usually happens within the consensus sequence of RRm6ACH (R = G or A, H = A, C, or U) and are mainly enriched in 3′ untranslated regions (3′ UTRs) proximal to the stop codon. The evidence demonstrates that N6-methyladenosine (m6A) plays an important role in numerous physiological and pathophysiological processes by influencing pre-mRNA processing, splicing (76), nuclear export (77), decay (78), and translation (79).

### m6A Methyltransferase (Writer)

The m6A writer, methyltransferase-like 3 and 14 proteins (METTL3 and METTL14) and their cofactors Wilms’ tumor 1-associating protein (WTAP) form a highly conserved m6A methyltransferase complex (MTC).

METTL3 is the main component of the MTC, and it can be found both in cytoplasm and in nucleus. Given the different localization, it functions distinctively (80). In the nucleus, the METTL3 can interact with the activated transcription factor SMAD2/3 to promote co-transcription of m6A on selective transcripts through the TGFβ signaling pathway (81). Moreover, METTL3 can bind to the transcription factor CEBPZ and aggregate at the transcription initiation site, promoting tumor development (82). In the cytoplasm, METTL3 acts as an m6A binding protein rather than a methylation enzyme. It can interact with eIF3h to recognize and bind to the 3’ end m6A site (83). It was showed that METTL3 is significantly overexpressed in PC and is related to poor prognosis. Knocking down METTL3 may reduce m6A levels and inhibits cell proliferation and invasion in PC (84). Furthermore, low METTL3 expression shows higher sensitivity to antitumor drugs such as gemcitabine, 5-fluorouracil, cisplatin and radiotherapy, suggesting that METTL3 could be a promising target for the treatment of PC patients (85).

METTL3 is the catalytic component in MTC, while METTL14 provides structural support for METTL3 close to its active site and also helps recognize METTL3 substrates (86). METTL14 are identified as a tumor suppressor in multiple types of cancers. However, METTL14 is overexpressed in PC. The upregulation of METTL14 can elevate the m6A level and decrease the expression of PERP, thereby promoting the proliferation and migration of PC cells both *in vivo* and *in vitro* (87). Loss of METTL14 can promote apoptosis induced by cisplatin in PC cells and enhance autophagy through an mTOR signaling-dependent pathway (88).

WTAP plays a crucial role in regulating the recruitment of the m6A methyltransferase complex to mRNA target proteins, acting as a regulatory subunit of the m6A MTC in the epitope regulation of RNA metabolism (89). WTAP also has a close relationship with tumor development. In PC, nuclear WTAP expression can be an independent prognostic indicator, where high expression is significantly correlated with poor overall survival and several pathological characteristics (90). Further studies show that WTAP can promote metastasis and suppress chemo-sensitivity to gemcitabine in PC cell lines *via* stabilizing Fak mRNA, and this function can be reversed by GSK2256098, a specific FAK inhibitor (91).

More co-factors of the m6A writer are also identified, such as viral-like m6A methyltransferase-associated protein (KIAAl429), RNA-binding motif protein 15/15B (RBM15/15B), and zinc finger CCCH domain protein 13 (ZC3H13). There are other independent m6A writers which do not work *via* the MTC, including methyltransferase-like 16 (METTL16), zinc finger CCHC-type containing 4 (ZCCHC4), and methyltransferase-like 5 (METTL5). However, the clinical impacts of them on PC are still unknown.

### m6A Demethylase (Eraser)

m6A demethylases include AlkB homolog 5 (ALKBH5) and fat mass and obesity-associated protein (FTO) (92). ALKBH5 decreased in PC cell lines. It can inhibit PC progression by demethylating the lncRNA KCNK15-AS1 (93). Besides, ALKBH5 could serve as a PC suppressor by regulating the post-transcriptional activation of PER1 in an m6A-YTHDF2-dependent manner (94). Through demethylation of m6A-modified Wnt inhibitory factor 1 (WIF-1) transcripts, ALKBH5 can impair the Wnt pathway and sensitize PC cells to chemotherapy (95).

In contrast, FTO promotes the growth of various cancer types. However, the role of FTO is not well understood in PC. Up to now, only one study reported that high expression of FTO in PC. Downregulation of FTO can inhibit proliferation of PC cells. Mechanistically, FTO can interact with the MYC proto-oncogene and bHLH transcription factor, thereby regulating its stability *via* decreased m6A modification (96).

### m6A Binding Protein (Reader)

The binding proteins of m6A include YTH structural domain proteins (YTHDF1, YTHDF2, YTHDF3, YTHDC1 and YTHDC2), members of the hnRNP family (hnRNPC and hnRNPA2B1), insulin-like growth factor 2 mRNA binding proteins (IGF2BP1, IGF2BP2, IGF2BP3), and eukaryotic initiation factor 3 (eIF3). Only YTHDF2 and IGF2BPs are involved in PC.

YTHDF2 expression is significantly upregulated in PC and related with poor survival in PC patients. Furthermore, YTHDF2 plays two different roles in cellular processes, including promoting proliferation and suppressing metastasis in PC cells, called the “migration-proliferation dichotomy”. Mechanistically, it is because downregulation of YTHDF2 can increase total YAP expression but suppress TGF-β/Smad signaling (97).

Several studies showed that high expression of IGF2BP1, IGF2BP2 and IGF2BP3 is associated with a poor prognosis in PC (98–100). In addition, IGF2BP2 is also found to be significantly upregulated in pancreatic intraepithelial neoplasia (PanIN), a vital precursor of PC, implying the ability of IGF2BP2 to be a diagnostic marker for early-stage PC (98). Functionally, IGF2BP2 can increase cell proliferation and metabolism in PC by directly binding and stabilizing GLUT1 mRNA (101). IGF2BPs can also interact with various ncRNAs in order to function in PC progression (102).

### 5-Methylcytosine (m5C)

m5C methylation is the process by which the 5th carbon atom (C5) on cytosine is modified by methylation. m5C can be found in tRNA, rRNA, mRNA, miRNA, or lncRNA. The distribution of m5C differs among RNAs of different species. For example, m5C is not present in bacterial tRNA and mRNA, while it is found in eukaryotic and prokaryotic tRNA and mRNA (103, 104). The distribution of this modification in mRNA is not random and mostly enriched in the 5 ’ and 3 ’ UTR and AGO protein binding sites (105, 106). In tRNA, m5C is mostly present at the junction of the variable arm and the T-stem spanning positions (107). As for rRNA, m5C was found in the anticodon loop, and identified only in 28S rRNA but not in 18S RNA (103). This modification is mostly located at the center of peptidyl transferase or at the interface of large and small subunits in rRNA (108), and the location of this modified position is quite conserved (109). In the field of ncRNAs, Hao yuan et al, constructs an mRNA–lncRNA co-expression network between m5C-related mRNAs and lncRNAs and indicates that the m5C-related lncRNA risk model can be a biomarker of prognosis and plays an essential role in regulating PC immune cell distribution (110).

### m5C Methyltransferase (Writer)

m5C methyltransferases include DNA methyltransferase 2(DNMT2) and NOL1/NOP2/sun (NSUN) subgroups, which use SAM as a methyl donor. The NSUN family includes NSUN1, NSUN2, NSUN3, NSUN4, NSUN5, NSUN6, and NSUN7. The only one related to PC is NSUN6, which is found to be decreased in PC tissues. NSUN6 can suppress the proliferation of PC cell lines both *in vivo* and *in vitro*. However, the expression level of NSUN6 in PC patients is tightly correlated with clinicopathologic parameters and overall survival, which could be a potential marker of PC (111). The opposite function is still unknown mechanistically and remains further investigated.

DNMT2 was first thought to be a DNA methyltransferase, but a study found that DNMT2 does not catalyze DNA methylation, rather interestingly catalyzes tRNA methylation at C38 (112). Nonetheless, the role of DNMT2 in PC needs to be clarified.

### m5C Binding Protein (Reader) and m5C Demethylase (Eraser)

Aly/REF export factor (ALYREF)is regarded as a specific binding protein for m5C-methylated mRNA. It can bind to m5C-enriched regions catalyzed by NSUN2, thereby regulating the out-of-nucleus movement of mRNA (113). Studies revealed that Y-box binding protein 1 (YBX1) may function as an m5C-binding protein which recognizes m5C binding sites and has a positive effect on mRNA stabilization and tumorigenesis (114, 115). The erasers of m5C methylation mainly include TET family members that identically act in DNA methylation. However, there is no study demonstrating the function of m5C readers and erasers in PC.

### N1-Methyladenosine (m1A)

m1A modification refers to the modification method of adding a methyl group to the first nitrogen atom (N1) of adenine and it is found in tRNA (116), rRNA (117), mRNA (118, 119) and mitochondrial transcripts (120). m1A modification plays a critical role in maintaining tRNA structure and translation (119, 120). It occurs at positions 9, 14 and 58 of the tRNA, where m1A58 is indispensable for the stability of the tRNA structure (121). Compared with other RNA modifications, the level of m1A remains low in mRNA. m1A modification in the 5 ’ cap region of mRNA may mediate translation (122). m1A modification of the mitochondrial mRNA coding region has been shown to affect the translation resistance of modified codons (120). m1A methyltransferases include tRNA methyltransferase (TRMTs) and NML. TRMT61A/6 is involved in modifying methylation at position 58 of tRNA in the cytoplasm (123), whereas the m1A methylation modification at positions 9 and 58 of mitochondrial tRNA is regulated by TRMT10C and TRMT61B (124, 125). In addition, m1A methylation was also found at position 1322 of 28s rRNA, catalyzed by nucleomethylin (NML) (126). ALKBH3 and ALKBH1 serve as erasers to remove m1A.

The significance of m1A for tumor development has been demonstrated in a variety of tumors. For example, knockdown of ALKBH3 can increase the m1A level of tRNA and decrease protein synthesis in cancer cells (127). ALKBH1 has also been confirmed to have an effect on tumorigenesis. In PC, it can promote cell proliferation through PI3K/AKT/mTOR and ErbB pathways (128).

However, compared with modifications such as m6A, our knowledge of m1A is far from adequate and the role of m1A in tumors needs to be established.

## Discussion

With recently rapid development of genome sequencing technologies, epigenetic changes have as essential events accounted for cancer progression and metastasis including PC. Methylation is the most common and important epigenetic modifications, including DNA methylation, histone methylation and RNA methylation, all of which are mediated by distinct writer enzymes, interpreted by reader proteins, and removed by eraser enzymes. Given the complicated mechanism of each modification in PC which has not been fully understood, the inconsistent evidence is reported. For example, in DNA methylation, hypermethylation catalyzed by writer can promote tumorigenesis; but in histone and RNA methylation, both writer and eraser can be oncogenes in PC, such as SMYD3 and KDM1, MELLT3 and FTO. This phenomenon is probably attributed to their distinct targets, in addition, other epigenetic regulators such as (de)acetylase, (de)phosphorylation and SUMO enzymes likely also participate in epigenetic modification, which co-occurs in the methylation modification, giving rise to divergent cellular outcomes. Therefore, the large effort is considerably taken into account in order to mechanistically illustrate the molecular network of epigenetic regulators that drive the malignancy of PC.

In the clinical aspects. there is strong evidence indicating the studies on methylation modification hold diagnostic and therapeutic value. Methylation signatures of cell free DNA *via* a non-invasive method can be tested for the identification of pre-neoplastic lesions and PC, assisting early diagnosis (129). At the present, this assay is only limited to DNA methylation, not for RNA or histone methylation because of the lack of detection technologies that analyze global methylation spectrum of RNAs or histones. The possibility in future of screening techniques detecting all of DNA, histone and RNA methylation will evidently assist the disease diagnosis. Some of methylation readers such as IGF2BP2 could serve as a diagnostic marker since its expression is significantly elevated in PanIN. Almost all regulators are reported to be independent prognosis indicators and are correlated with clinical outcomes. In addition, the therapeutic application has recently received significant attention, as those epigenetic regulators can be potential targets for anti-cancer therapy. Distinct inhibitors of epigenetic enzymes mainly targeting at DNA methyltransferases (DNMTs), histone methyltransferases (HMTs) and histone demethylases (HDMs) exhibit strong ability to interfere histone and DNA methylation process, thus some of these inhibitors have been used in clinical trials. Meanwhile, there have been many clinical studies on methylation in liver cancer, colorectal cancer which can provide ideas for the treatment of PC. Therefore, the discovery of methylation mechanisms and development of advanced technologies will be beneficial to the clinical diagnosis and treatment.

In conclusion, with recently intense focus on epigenetic methylation of varied key molecules that mediate pathogenesis of PC, the novel discovery including aberrant expression of dysfunctional factors as potential biomarkers, therapeutic targets, and methylation blockers will offer great value to assist the early diagnosis, prediction of recurrence and prognosis, and targeted therapy of PC. For example, many studies have identified the sensitivity and specificity of single locus promoter methylation in tissue of PC, however, substantial large sample volume from multiple cancer centers with a variety of disease stages is essential to firmly establish the specific methylated single locus as a diagnostic or prognostic marker for PC. As the heterogeneity of PC, multigene methylations that regulate distinct signaling pathways in individual components of the tumor microenvironment coordinately promote the malignant transformation of PC. Therefore, the growing research will give rise to solid evidence favorable to lay the foundation of creating novel means to treat this lethal disorder. As expected, revealing the methylation function will encourage researchers to extensively focus on the mechanistic study, ultimately offering both potential biomarkers valuable for diagnosis and therapeutic strategy to treat this devastated disorder.

## Author Contributions

YZ, MY, and SW drafted the manuscript. JZ and YSL coordinated and edited the drafting of the manuscript. SJA, RS, and YBL revised and edited the final version of the manuscript. All authors contributed to the article and approved the submitted version.

## Funding

This work was supported by the National Natural Science Foundation of China (32130036, 81874181).

## Conflict of Interest

The authors declare that the research was conducted in the absence of any commercial or financial relationships that could be construed as a potential conflict of interest.

## Publisher’s Note

All claims expressed in this article are solely those of the authors and do not necessarily represent those of their affiliated organizations, or those of the publisher, the editors and the reviewers. Any product that may be evaluated in this article, or claim that may be made by its manufacturer, is not guaranteed or endorsed by the publisher.
